# Natriuretic peptide-induced hyponatremia in a patient with left atrial myxoma

**DOI:** 10.1186/cc9788

**Published:** 2011-03-11

**Authors:** D Ramnarain, N Mehra

**Affiliations:** 1 St Elisabeth Ziekenhuis, Tilburg, the Netherlands; 2 University Medical Centre Utrecht, the Netherlands

## Introduction

In addition to the renin-angiotensin-aldosterone system, natriuretic peptides act as regulators of blood pressure. Natriuretic peptides increase sodium and water excretion, increase the glomerular filtration rate, and are vasodilatators. We report a case in which a large atrial myxoma induced overproduction of natriuretic peptides, causing clinically relevant hyponatriemia, hypotension and polyuria.

## Methods

We present a 74-year-old Caucasian female who was referred by her cardiologist for resection of a large left atrial myxoma.

## Results

The patient's medical history was unremarkable except for irritable bowel syndrome, mild hypertension, and recently paroxysmal atrial fibrillation due to growth of her myxoma. A month preoperatively a laboratory study indicated a mild hyponatriemia. Clinical investigation postoperatively showed a hypovolemic patient, with a blood pressure of 85/32 mmHg, a heart rate of 54 bpm, and CVD <5 mmHg. There were no signs of heart failure. Urine production was 200 ml/hour without any diuretic therapy, and remained high during 2 days after surgery. Laboratory investigation showed increased ANP levels during the patient's stay. Sodium was 129 mmol/l and decreased to 127 mmol/l, GFR >60 ml/minute, serum osmolarity was 262 mOsmol/kg. Natriuresis was 175 mmol/l, urine osmolarity was 563 mOsmol/kg. Pathological examination showed a large myxoma, connected to the fossa ovalis (4.3 × 4.5 × 3 cm). On the third day her urine production decreased to 70 ml/hour. Hyponatremia persisted and 10 days later her sodium level normalised.

## Conclusions

We propose a mechanism of hyponatremia caused by overproduction of physiologically active natriuretic peptides by atrial stretch and ventricular stretch caused by a large intracardial tumour. Atrial stretch releases ANP and ventricular stretch releases BNP from myocardial cells. Normally increased intracardial stretch implies a volume expansion, and release of natriuretic peptides act to regulate blood pressure by increasing sodium and water excretion. A large intracardial tumour attached to the embryonic remnant of the fossa ovalis caused intracardial stretch, mimicking a hypervolemic state. Overproduction of natriuretic peptides is seen in different clinical aetiologies such as intracerebral haemorrhage, lung cancer and pneumonia, linking natriuretic peptides to cerebral salt wasting and SIADH. We provide evidence of a rare cause of hyponatremia and polyuria caused by overproduction of the physiological natriuretic peptide system by a large myxoma.

